# Persisting post-infection symptoms 2 years after a large waterborne outbreak of *Cryptosporidium hominis* in northern Sweden

**DOI:** 10.1186/s13104-018-3721-y

**Published:** 2018-08-30

**Authors:** Mikael Lilja, Micael Widerström, Johan Lindh

**Affiliations:** 1 Department of Public Health and Clinical Medicine, Unit of Clinical Research Center-Östersund, Umeå University, Umeå, Sweden; 2 Department of Clinical Microbiology, Unit of Communicable Disease Control and Prevention-Östersund, Umeå University, Umeå, Sweden; 3 Department of Cell and Molecular Biology, Microbiology, Uppsala University, Box 256, 751 05 Uppsala, Sweden

**Keywords:** *Cryptosporidium*, Sequelae, Diarrhea

## Abstract

**Objectives:**

In 2010–2011, a large waterborne outbreak of *Cryptosporidium hominis* affected the city of Östersund in Sweden. Previous findings had suggested that gastrointestinal symptoms can persist for up to 11 months after the initial infection. Here we investigated whether the parasite could cause sequelae in infected individuals up to 28 months after the outbreak. We compared cases linked to the outbreak and the previous follow-up study with non-cases regarding symptoms present up to 28 months after the initial infection. We investigated whether cases were more likely to report a list of symptoms at follow-up compared to non-cases, calculating odds ratio and 95% confidence interval obtained through logistic regression.

**Results:**

A total of 559 individuals (215 cases) were included in the study. Forty-eight percent of the outbreak cases reported symptoms at follow-up. Compared to non-cases, cases were more likely to report watery diarrhea, diarrhea, abdominal pain, fatigue, nausea, headache, or joint stiffness/pain/discomfort at follow-up after adjusting for age and sex. Our findings suggest that gastrointestinal symptoms and joint pain can persist several years after the initial *Cryptosporidium* infection and should be regarded as a potential cause of unexplained gastrointestinal symptoms or joint pain in people who have had this infection.

**Electronic supplementary material:**

The online version of this article (10.1186/s13104-018-3721-y) contains supplementary material, which is available to authorized users.

## Introduction

*Cryptosporidium* is a protozoan parasite that can cause gastrointestinal illness in humans and animals [1]. Cryptosporidiosis is transmitted mainly by the fecal–oral route, primarily through oocyst-contaminated water or food, or by direct contact with an infected person or animal [2]. Cryptosporidiosis occurs worldwide and in all age groups [1], although children are most frequently and most severely affected, particularly during the first year of life [3].

In otherwise healthy individuals, infection with *Cryptosporidium* causes gastrointestinal illness. The most common symptoms are diarrhea, watery diarrhea, nausea, vomiting, fever, and abdominal pain [1], but infections can be asymptomatic [2]. Relapse of diarrhea more than 2 days after the end of initial diarrheal disease occurs in over a third of cases [4, 5]. Evidence of long-term gastrointestinal symptoms after initial infection is limited, although gastrointestinal symptoms persisting up to 3 months [6], up to 11 months, and up to 3 years [7–9] after primary infection have been reported. Similar to other gastrointestinal pathogens, *Cryptosporidium* infection can also cause reactive arthropathy. Such *Cryptosporidium*-induced joint disease was first described in case reports in the 1980s [10, 11], and more recent epidemiological studies have suggested the presence of joint-related symptoms for up to 3 months [6] and up to 11 months [9, 12] after the original infection.

In 2010–2011, two large waterborne outbreaks caused by the same *Cryptosporidium* genotype (*C. hominis* IbA10G2) affected two cities in the northern part of Sweden [5]: first in November 2010 in Östersund, and 2 months later in Skellefteå. A follow-up evaluation of the affected population revealed that outbreak cases were more likely to have gastrointestinal and joint-related symptoms up to 11 months after the initial infection [9]. We performed an additional follow-up investigation of the study population in Östersund to ascertain whether the symptoms were persisting 2 years after the outbreak occurred.

## Main text

### Methods

In the present investigation, data collected in the outbreak cohort studies were used to determine case status (presence of symptoms during the outbreak and duration of symptoms), apply exclusion criteria (chronic diseases), and perform a non-response analysis.

#### Study population

In mid-January 2011, 2 months after the onset of the *Cryptosporidium* outbreak in Östersund, 1524 people (2.5%) in a random sample of individuals living in that city were invited to take part in a retrospective cohort study to assess the magnitude of the outbreak, clinical characteristics, and risk factors for cryptosporidiosis [5]. Eight hundred seventy-two of those subjects also took part in a questionnaire-based follow-up investigation [9], and, 25 January 2013, we invited those respondents to complete another follow-up questionnaire regarding post-infection symptoms during the 3 months preceding the day of answering the questionnaire. Responses were recorded until 25 February 2013, and hence the period of recorded post-infection symptoms in the Östersund study was 25 October 2012 to 25 February 2013.

#### Case definition

We determined outbreak case status based on clinical characteristics reported in the outbreak questionnaire. We defined an outbreak case of *Cryptosporidium* infection as a respondent who reported new episodes of diarrhea (more than three loose stools in 1 day) and/or watery diarrhea and a duration of gastrointestinal symptoms of at least 4 days during the outbreak period (1 November 2010 to 31 January 2011). We defined respondents not fitting the case definition as being non-cases during the outbreaks.

#### Data collection

The follow-up questionnaire together with a letter explaining the study and a pre-paid envelope for return of the completed questionnaire to the Public Health Agency of Sweden were sent to the study population by ordinary mail. Parents or guardians were asked to respond for children < 15 years of age. The questionnaire included items about symptoms present during the follow-up period (as defined above): loss of appetite, weight loss, diarrhea, watery diarrhea, bloody diarrhea, abdominal pain, headache, eye pain, fatigue, stiff joints, joint pain, swollen joints, joint discomfort, nausea, vomiting, and a blank area where the respondent could describe any other symptoms (Additional files 1, 2). All of the questionnaires that were returned were optically scanned and transformed into an electronic database.

#### Exclusion criteria

To avoid potential misclassification of case status, such as bias at follow-up linked to chronic gastrointestinal symptoms induced by causes other than *Cryptosporidium* infection, we excluded study participants that had reported a diagnosis of irritable bowel syndrome or inflammatory bowel disease on the outbreak questionnaire.

#### Data analysis

We described the study populations in terms of symptoms present at follow-up, age group, and sex in relation to outbreak case status. Logistic regression models, with symptoms at follow-up as outcome and case status as explanatory factor, were used to calculate odds ratios (ORs) and 95% confidence intervals (CIs). We included age and sex in the model to adjust for possible confounding effects of these variables. To determine whether associations between symptoms at follow-up and case status differed between age groups, we also built logistic regression models for each age group, and we included sex in those models to adjust for its potential confounding effect. Observations with missing values were excluded from the analysis.

#### Non-response analysis

We examined whether sex, age, postal code, and case status of the members of the study population were associated with non-response by calculating OR and 95% CI using logistic regression modeling.

#### Microbiological investigation

In the questionnaire, we asked the study participants if they were willing to provide a fecal sample, and those who agreed to do so were sent a collection kit that included a sampling vessel and instructions on how to collect fecal material. Fecal samples from the study group were analyzed for *Cryptosporidium* using a standard concentration technique followed by modified Ziehl–Neelsen staining [13].

### Results

#### Study population

A total of 588 individuals responded to the follow-up questionnaire, representing a response rate of 67%. Twenty-nine of those individuals (13 cases and 16 non-cases) met the exclusion criteria and hence were not included in the evaluation. Thus 559 individuals were included in the analysis (215 cases and 344 non-cases). The characteristics of the study population are presented in Table 1. The median age was 41 (range 3–79) years among cases and 56 (range 3–95) years among non-cases.

**Table 1:** Demographic characteristics of the study population presented by outbreak case status, Sweden 2013

Characteristic	Cases n (%)	Non-cases n (%)	Total n (%)
Sex			
Women	123 (57)	190 (55)	313 (56)
Men	92 (43)	154 (45)	246 (44)
Age group (years)			
0–5	28 (13)	20 (6)	48 (9)
6–15	28 (13)	26 (8)	54 (10)
16–40	48 (22)	66 (19)	114 (20)
41–65	80 (37)	120 (35)	200 (36)
> 65	31 (14)	112 (33)	143 (26)
Total	215 (100)	344 (100)	559 (100)

#### Symptoms during the follow-up period

Forty-eight percent of cases reported symptoms at follow-up, most frequently headache, fatigue, abdominal pain, and nausea (Table 2). Compared to non-cases, the cases were more likely to report watery diarrhea, diarrhea, abdominal pain, stiff joints, joint pain, joint discomfort, fatigue, nausea, and headache at follow-up after adjusting for age and sex (Table 2). The likelihood of cases reporting symptoms at follow-up differed between age groups: joint pain (OR 13.2, CI 2.8–61.9) and nausea (OR 2.7, CI 1.2–6.0) were associated only with the 16–40-year age group; diarrhea (OR 3.9, CI 1.1–14.3) was associated only with the > 65-year age group; and headache (OR 4.0, CI 1.3–13.1) was associated only with the 6–15-year-old age group. No significant associations were found in the youngest age group.

**Table 2:** Symptoms reported at follow-up by questionnaire and association with case status, Sweden 2013

Symptom	Total			0–5 years	6–15 years	16–40 years	41–65 years	> 65 years
	n = 559			n = 48	n = 54	n = 114	n = 200	n = 143
	Cases n (%)	Non-cases n (%)	OR^a^ (95% CI)	OR^b^ (95% CI)	OR^b^ (95% CI)	OR^b^ (95% CI)	OR^b^ (95% CI)	OR^b^ (95% CI)
Weight loss	9 (4)	14 (4)	0.9 (0.4–2.2)	0.6 (0.1–5.6)	–	1.7 (0.4–8.4)	0.9 (0.2*–*3.8)	–
Watery diarrhea	36 (17)	26 (8)	*2.0 (1.1–3.4)*	1.4 (0.3–5.5)	4.5 (0.4–44.5)	1.6 (0.6–4.2)	2.8 (1.0*–*7.5)	1.3 (0.1–13.1)
Diarrhea	53 (25)	45 (13)	*1.9 (1.7–2.9)*	0.9 (0.3–3.3)	0.9 (0.2–4.1)	2.4 (1.0–5.8)	2.1 (1.0–4.6)	*3.9 (1.1–14.3)*
Abdominal pain	61 (29)	43 (13)	*2.3 (1.5–3.7)*	4.3 (0.9–22)	1.4 (0.4–4.5)	*3.1 (1.3–7.1)*	1.8 (0.8–4.1)	*4.3 (1.2–14.7)*
Stiff joints	44 (21)	38 (11)	*2.8 (1.7–4.6)*	–	–	*4.0 (1.1–14.1)*	*2.1 (1.1 –4.4)*	*3.1 (1.2–7.9)*
Joint pain	48 (23)	44 (13)	*2.2 (1.5–3.7)*	*–*	0.5 (0.1–2.5)	*13.2 (2.8–61.9)*	1.8 (0.9–3.6)	2.1 (0.8–5.5)
Loss of appetite	30 (14)	39 (11)	1.0 (0.6–1.7)	0.4 (0.1–1.7)	1.3 (0.3–6.7)	1.5 (0.6–3.7)	1.0 (0.4–2.6)	–
Fatigue	80 (38)	84 (25)	*1.7 (1.1–2.5)*	1.2 (0.3–4.4)	0.8 (0.2–2.8)	*3.0 (1.4–6.5)*	*2.0 (1.1–3.7)*	0.8 (0.3–2.5)
Nausea	60 (29)	49 (15)	*2.0 (1.3–3.0)*	1.2 (0.3–4.7)	0.8 (0.2–3.2)	*2.7 (1.2–6.0)*	2.0 (0.9–4.4)	2.2 (0.5–9.9)
Headache	95 (45)	82 (24)	*2.4 (1.7–3.5)*	3 (0.5–17.1)	*4 (1.3–13.1)*	*3.2 (1.4–7.3)*	*2.4 (1.3–4.4)*	1.0 (0.3–3.0)
Joint discomfort	40 (19)	44 (13)	*2.0 (1.2–3.3)*	–	–	*6.1 (1.8–20.3)*	1.6 (0.8–3.3)	1.7 (0.6–4.5)
Ocular pain	38 (18)	42 (12)	1.5 (0.9–2.4)	0.7 (0.1–5.1)	1.1 (0.2–4.7)	1.4 (0.6–3.7)	1.4 (0.6–3.1)	2.8 (0.9–8.8)
Swollen joints	13 (6)	20 (6)	1.2 (0.5–2.6)	–	–	7.7 (0.9–68.0)	0.5 (0.1–1.5)	2.4 (0.6–9.1)
Vomiting	34 (16)	26 (8)	1.6 (0.9–2.8)	1.0 (0.3–3.4)	1.7 (0.3–10.3)	1.5 (0.6–3.7)	1.5 (0.4–5.5)	–

Italics indicates significant at 0.05 level

^a^Adjusted for age and sex

^b^Adjusted for sex

#### Non-response analysis

Compared to the reference age group 0–5 years, the older age groups were more likely to respond to the follow-up questionnaire: 15–40 years old, OR 2.9 (CI 1.9–4.5); 40–65 years, OR 6.5 (CI 4.3–10.1); > 65 years, OR 11.4 (CI 6.9–19.1). Sex, postal code, or case status did not explain non-response in any of the studies.

#### Microbiological investigation

A total of 183 subjects provided fecal samples (119 adults and 64 children), and 138 (73%) of those individuals were defined as previous cases. *Cryptosporidium* was not detected in any of the 183 samples.

### Discussion

We investigated post-infection health consequences following a waterborne *C. hominis* outbreak in northern Sweden based on data from a random sample of the population. We have previously reported that outbreak cases were more likely to have gastrointestinal and joint-related symptoms and had a larger number of symptoms up to 11 months after the initial infection compared to non-cases [9]. The present results suggest that the same symptoms are still more common among cases compared to non-cases up to 2 years after the outbreak. The middle-aged subjects in the study population were most affected, particularly by joint-related symptoms. It should also be noted that the cases were negative for *Cryptosporidium* infection 2 years after the outbreak. Our findings imply that the health consequences of *Cryptosporidium* infection persist even longer than indicated in previous reports [6, 10], although gastrointestinal symptoms have been described to continue for 24–36 months in up to eight of 53 *Cryptosporidium* cases in Stockholm [7]. Similar long-term effects following initial infection have also been recorded for other gastrointestinal parasites, e.g. *Giardia duodenalis* [14, 15].

The attack rate during the Östersund outbreak was highest in 20–29-year-olds (58%), followed by 40–49-year-olds (55%) and 0–9-year-olds (51%). Our follow-up data show that the outbreak cases up to age 15 years showed no significant increase in post-infection gastrointestinal or joint-related symptoms 2 years after the outbreak. However, the 16–40-year-old cases were more likely to report symptoms at follow up, especially joint-related symptoms. This suggests that the overall impact of the outbreak on the health of the population was most pronounced among young adults and adults but was more limited among younger individuals. A possible explanation for this finding is that stress influenced the symptom outcomes, because levels of stress hormones can be expected to vary more extensively in adults and young adults than in children [16].

## Limitations

Our findings derive from a waterborne outbreak in Sweden in which 27,000 people were estimated to be infected. Only a small proportion of the cases were tested and laboratory confirmed, and no additional sampling was performed on the study population during the outbreak to verify case status. This lack of laboratory confirmation is a limitation in the current analysis, leading to a number of minor concerns regarding misclassification of case status, as outlined below.

First, some of the subjects considered as cases in our study may have actually had a norovirus infection (or infection with some other gastrointestinal pathogen) rather than cryptosporidiosis. However, our case definition required duration of symptoms of > 4 days, a period that is longer than what is normally expected for a norovirus infection [17], which probably excluded potential norovirus cases. In addition, *Cryptosporidium* was detected in the drinking water during the outbreak, suggesting that cases of gastroenteritis during this time were no doubt the result of *Cryptosporidium* infection. The 149 laboratory-confirmed cases of cryptosporidiosis related to the outbreak in Östersund were all negative for other gastrointestinal pathogens, including norovirus [5].

Second, individuals with chronic intermittent diarrhea were probably classified as cases during the outbreak, and it is also likely that they reported similar symptoms at follow-up. We attempted to minimize this effect by excluding cases diagnosed with irritable bowel syndrome or inflammatory bowel disease. Nonetheless, it is possible that some individuals who had undiagnosed persisting gastrointestinal symptoms, and reported them as new episodes of diarrhea, were included in our studies and were defined as outbreak cases and as cases with symptoms at follow-up, resulting in an overestimation of the associations.

Third, any asymptomatic and mild cases that suffered from *Cryptosporidium*-related post-infection symptoms probably contributed to the prevalence of symptoms in the non-case group in the present follow-up, and hence diluted the true association, indicating that our findings may be underestimated.

It is also possible that our results were influenced by recall bias and non-response bias. Recall bias might have affected cases and non-cases differently, because cases may have been more self-observant due to the initial infection and therefore to a greater extent prone to remember more symptoms. However, non-response analysis did not suggest that cases were more likely to respond. Furthermore, some vague symptoms could plausible be of a more psychosomatic nature. However, for the cases reporting these, they are real and will have bearing of their lives.

## Additional files

**Additional file 1.** Questionnaire Swedish original version.

**Additional file 2.** Questionnaire, translated version to English.

## Abbreviations

CI: confidence interval
OR: odd ratio
