# Supplementary material for: Persisting post-infection symptoms 2 years after a large waterborne outbreak of Cryptosporidium hominis in northern Sweden
Source: BMC Res Notes. 2018 Aug 30;11:625. doi: 10.1186/s13104-018-3721-y (PMC6117891; doi:10.1186/s13104-018-3721-y)
Supplement: Supplementary file 1 — Additional file 1. Questionnaire Swedish original version. [file 13104_2018_3721_MOESM1_ESM.pdf]

Enkät uppföljning efter Cryptosporidium-utbrottet vintern 2010/2011. (vuxna)

(Sätt ett kryss i rutorna och använd kulspetspenna)

Födelseår: ☐ (åååå) **Jag är:** ☐ man ☐ kvinna

1. **Har du de senast tre månaderna haft några av följande symtom**

	1-2 dagar	*2-6 dagar*	*1 vecka eller mer*	*Nej*
**- diarré med 3 eller fler lösa avföringar per dag**	☐	☐	☐	☐
**- vattning diarré**	☐	☐	☐	☐
**- blod i diarrén**	☐	☐	☐	☐
**- buksmärtor/magkramper**	☐	☐	☐	☐
**- kräkningar**	☐	☐	☐	☐
**- illamående**	☐	☐	☐	☐
**- huvudvärk**	☐	☐	☐	☐
**- ont i ögonen**	☐	☐	☐	☐
**- trötthet/utmattning**	☐	☐	☐	☐
**- aptitlöshet**	☐	☐	☐	☐
**- viktnedgång**	☐	☐	☐	☐
**om viktnedgång:** ☐ (antal kilo)				
**- ledbesvär**	☐	☐	☐	☐
**- stela leder**	☐	☐	☐	☐
**- ledvärk**	☐	☐	☐	☐
**- svullna leder**	☐	☐	☐	☐

2. **Om du har haft några av symtomen i fråga 1, hur sjuk har du känt dig?**
Sätt en kryss i den ruta som stämmer bäst överens för dig. *0 = inte alls sjuk och 10 = mycket sjuk.*

0	*1*	*2*	*3*	*4*	*5*	*6*	*7*	*8*	*9*	*10*
☐	☐	☐	☐	☐	☐	☐	☐	☐	☐	☐

01

3. **Har du de senaste 3 månaderna varit hemma från arbete/studier på grund av att du haft symtom enligt fråga 1?**

Ja ☐ *Nej* ☐

Om ja, hur länge? ☐ (antal dagar)

4. **Har du besökt sjukvården de senaste 3 månaderna för den symptomen i fråga 1?**
Kryssa i ett eller flera alternativ.
Kommentera gärna vid fråga 10.

☐ nej
☐ ja, hälsocentral
☐ ja, sjukhus

5. **Har du något/några av följande besvär eller sjukdomar:**

	Ja	*Nej*
**- magsårsbesvär**	☐	☐
**- irritabel tarm** (IBS)	☐	☐
**- inflammatorisk tarmsjukdom** (Ulcerös kolit eller Crohns sjukdom)	☐	☐
**- glutenintolerans**	☐	☐
**- laktosintolerans**	☐	☐
**- andra långvariga tarmbesvär**	☐	☐
**- diabetes**	☐	☐
**- KOL/astma**	☐	☐
**- hjärtsvikt**	☐	☐
**- reumatisk ledsjukdom**	☐	☐
**- cancer**	☐	☐

6. **Behandlas du nu med något/några av följande läkemedel?**

	Ja	*Nej*
**- medicin mot magsår/sura uppstötningar** (t ex Omeprazol, Losec, Nexium)	☐	☐
**- kortisontabletter**	☐	☐
**- cellgifter eller andra läkemedel som nedsätter immunförsvaret?**	☐	☐

7. **Tror du själv att du har några aktuella symptom / besvär orsakade av Cryptosporidie-infektion?**

Ja ☐ *Nej* ☐

8. **Känner du dig orolig för din hälsa pga Cryptosporidium?**
Sätt en kryss i den ruta som stämmer bäst överens för dig. *0 = inte alls sjuk och 10 = mycket sjuk.*

0	*1*	*2*	*3*	*4*	*5*	*6*	*7*	*8*	*9*	*10*
☐	☐	☐	☐	☐	☐	☐	☐	☐	☐	☐

9a. **Skulle du kunna tänka dig att medverka i en fördjupad enkät där vi mer i detalj frågar om hur du mår. Denna enkät tar cirka 10 minuter att fylla i.**

Ja ☐ *Nej* ☐

9b. **Skulle du kunna tänka dig lämna ett avföringsprov**

☐ ☐

10. **Är det något du vill tillägga gällande ditt hälsotillstånd som du tror beror på att du var i Östersund under parasitutbrottet vintern 2010?**

....

....

....

....

....

....

....

....

....

....

....

....

....

....

....

....

Tack för att du tog dig tid att besvara frågorna!

Vi vill gärna att du skickar ditt svar så snart möjligt i bifogat svarskuvert.
