# Supplementary material for: Persisting post-infection symptoms 2 years after a large waterborne outbreak of Cryptosporidium hominis in northern Sweden
Source: BMC Res Notes. 2018 Aug 30;11:625. doi: 10.1186/s13104-018-3721-y (PMC6117891; doi:10.1186/s13104-018-3721-y)
Supplement: Supplementary file 2 — Additional file 2. Questionnaire, translated version to English. [file 13104_2018_3721_MOESM2_ESM.docx]

**Surveys follow-up after the Cryptosporidium outbreak winter 2010/2011. (Adults)**

(Tick the boxes and use the ballpoint pen)

Year of birth I am male female

**1. Have you had any of the following**

**symptoms in the last three months?** *1-2 days 2-6 days 1week or* *more No*

**- diarrhea with 3 or more loose**

**stools per day**

**- watering diarrhea**

**- blood in the diarrhea**

**- abdominal pain / abdominal cramps**

**- vomiting**

**- nausea**

**- headache**

**- pain in the eyes**

**- fatigue**

**- loss of appetite**

**- weight loss**

**If wright loss. Kg**

**- joint discomfort**

**- stiff joints**

**- joint pain**

**- swollen joints**

**2. If you have had any of the symptoms in question 1, how ill have you felt?**

Put a cross in the box that best suits you. 0 = not at all ill and 10 = very ill.

**3. Have you been home from work / studies for the last 3 months because you had symptoms in Question 1?**

**If yes, how long?**

**4. Have you visited a care center during the last** no, yes, health center,

**3 months for the symptom in question 1?** yes, hospital

**5. Do you have any of the following symptoms or illnesses: yes no**

**- stomach ulcer**

**- irritable bowel (IBS)**

**- inflammatory bowel disease**

**(Ulcerative Colitis or Crohn's Disease)**

**- gluten intolerance**

**- lactose intolerance**

**- other long-term intestinal disorders**

**- diabetes**

**- COPD / asthma**

**- heart failure**

**- rheumatic joint disease**

**- cancer**

**6. Are you treated with any of the following medicines? Yes no**

**- medicine for gastric ulcer / acid rejections**

(e.g. Omeprazol, Losec, Nexium)

**- cortisone**

**- cell cures or other drugs that reduce the immune system?**

**7. Do you think you have any current symptoms / symptoms caused by Cryptosporidie infection?**

**8. Do you feel worried about your health because of Cryptosporidium?**

Put a cross in the box that best suits you. 0 = not at all ill and 10 = very ill.

**9a. Would you like to participate in an in-depth survey, where we ask you more in detail about how you feel. This survey takes about 10 minutes to fill in.** yes no

**9b Could you leave a stool sample** yes no

**10. Is there anything you want to add regarding your health condition that you think is due to the parasite outbreak in winter 2010?**

Answer in free text
